# Reporting two hemoglobin J Iran cases, molecular follow-up, or is it insignificant? 

**DOI:** 10.22088/cjim.14.3.586

**Published:** 2023

**Authors:** Amin Solouki, Tahereh Manoochehrabadi, Farzaneh Korani, Mohammadreza Farshchi

**Affiliations:** 1 Department of Hematology and Blood Banking, School of Allied Medical Sciences, Shahid Beheshti University of Medical Sciences, Tehran, Iran; 2 Department of Tissue Engineering & Regenerative Medicine, Faculty of Advanced Technologies in Medicine, Iran University of Medical Sciences (IUMS), Tehran, Iran; 3 Hematology and Electrophoresis Department, Kermanshah Reference Laboratory, Kermanshah, Iran

**Keywords:** Hemoglobinopathy, Thalassemia, Hemoglobin J, Fast hemoglobin, Molecular diagnosis

## Abstract

**Background:**

Hemoglobin J is one of the fast hemoglobin that has a more negative charge due to β77His→Asp substitution. Acquisition of this hemoglobin is not associated with any specific clinical sign, but the combination of this hemoglobinopathy with beta-thalassemia and other hemoglobinopathies can cause challenges.

**Case Presentation:**

In this article, two cases with hemoglobin J are introduced; the first patient for premarital testing and the other for his fatigue. The hemoglobin electrophoresis was done by Sebia capillary zone electrophoresis and Hb J as heterozygote and homozygote were determined.

**Conclusion:**

It must be noted that although this hemoglobinopathy is not related to any problem alone but could be confusing in combination with other hemoglobinopathies or thalassemia. In this paper, these two cases are introduced and an attempt was made to investigate the importance of molecular follow-up.

Hemoglobinopathies are disorders caused by various mutations in the alpha or beta hemoglobin genes. The importance of hemoglobinopathies is in the affinity and structural change of hemoglobin )Hb) and the combination with Sickle cell anemia could be problematic (1). The β-chain has a negative charge and easily assembled with the positively charged α-chain to form hemoglobin tetramer. Chromosome 11 has two β genes, and the α-genes are located on chromosome 16, which β-globin chains are in balance with the four α globin chains. Qualitative mutations (hemoglobinopathies) in the β-chain can change its charge. In β-chain hemoglobinopathies, the Hb variant is usually lower than the normal Hb because most beta variants make the beta more positive. For example, in a patient with sickle cell trait, HbA content is higher than HbS (2). Hemoglobin movement on electrophoresis depends on its charge and fast hemoglobins are a group of hemoglobin that move faster to the anode due to changes in their charge (3,4). The hemoglobin J family, such as Baltimore, Bangkok, and Iran, belong to this group. Hemoglobin J Iran (β77His→Asp) was first reported in 1987, which has a more negative charge (5). This hemoglobin could interfere with the diagnosis of clinically important hemoglobins because in the routine state, in other hemoglobinopathies the amount of normal hemoglobin is higher than mutant hemoglobin, but in this hemoglobinopathy due to more negative charge of Hb J, this is not true. According to the literature review, the prevalence of this disease is not determined, but it is one of the rare hemoglobinopathies. In this paper, 2 cases with HbJ will be presented and discussed.

## Case Presentation

These patients have been selected from those who referred to Kermanshah Reference Laboratory, Kermanshah, Iran. They have signed an informed consent and this report is under the Iran National Committee for Ethics in Biomedical Research, code number IR.GMU.REC.1401.013.

**Figure 1:**
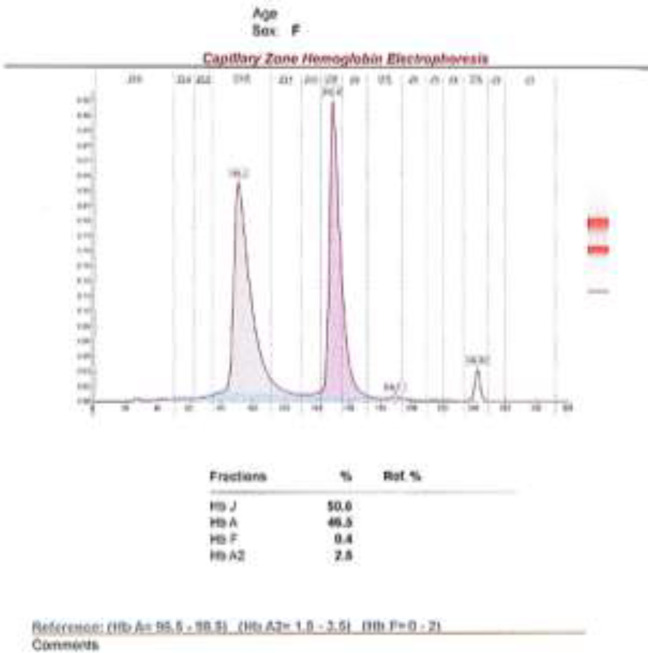
Capillary zone electrophoresis of case 1, Hb A: 46.5, Hb J: 50.6%, Hb F:0.4%, Hb A2:2.5%

**Case 1**: A 31-year-old female who was referred to Kermanshah Reference Laboratory for premarital screening program. Her CBC results performed with the Sysmex kx21 automated cell counter indicated that the red blood cell indices were normal. Also, examination of the iron profile showed that iron reserves were normal. A summary of her CBC is shown in table 1. Electrophoresis was performed by Sebia capillary zone electrophoresis and the results showed the presence of hemoglobin J as heterozygous. The results are shown in table 1. Her electrophoresis result is shown in figure 1.

**Figure 2:**
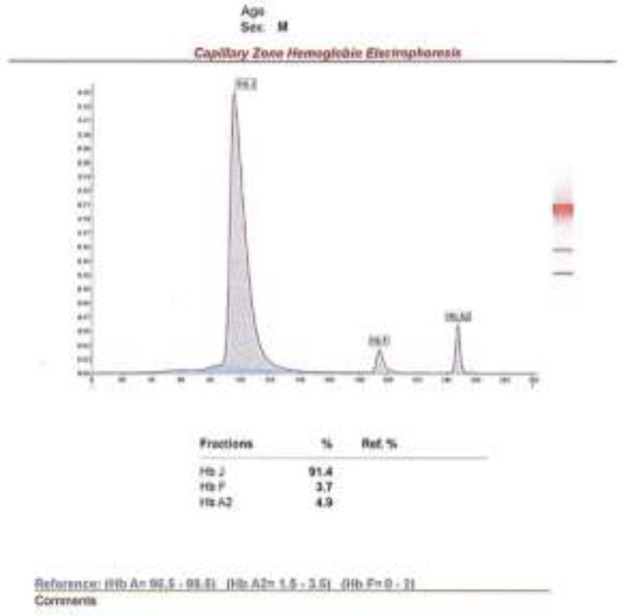
Capillary zone electrophoresis of case 2, Hb J: 91.4%, Hb F:3.7%, Hb A2:4.9%

**Case 2**: A 45-year-old man who underwent CBC testing and electrophoresis with those instruments for evaluation of his fatigue and presence of anemia. He was diagnosed with microcytic hypochromic anemia and his red blood cell indices were thalassemic. His electrophoresis results showed the absence of HbA and elevation of HbA2. The electrophoresis result of case 2 is shown in figure 2 and the CBC and electrophoresis results are also summarized in table 1.

**Table1:** CBC and Electrophoresis results

**Case 2/Male**	**Case 1/Female**	**CBC**
5.68	4.66	**RBC (×10** ^12^ ** /L)**
10.8	13.4	**HB(g/dL)**
34.6	40.5	**HCT (%)**
61.0	87.2	**MCV (fL)**
19.0	28.7	**MCH (pg)**
31.2	33.0	**MCHC(g/dL)**
Case 2	Case 1	**Electrophoresis**
0%	46.5%	**HbA**
91.4%	50.6%	**HbJ**
3.7%	0.4%	**HbF**
4.9%	2.5%	**HbA2**

RBC: Red Blood Cell, Hb: Hemoglobin, HCT: Hematocrit, MCV: Mean corpuscular volume, MCH: Mean corpuscular hemoglobin, MCHC: Mean corpuscular hemoglobin concentration

## Discussion

Hemoglobin J Iran is due to β77His→Asp, and this substitution causes a more negative charge for the β_J_ chain. As a result, in the case of heterozygotes with normal β-chain, the amount of variant hemoglobin is higher than HbA, which was observed in case 1(this differs from other variants such as sickle cell which variant Hb is lower than the normal one) (6). In these patients (heterozygous), the indices are generally normal, and HbA2 does not increase. Considering that no specific clinical signs have been observed in this patient (case 1), it can be assumed that hemoglobin J Iran does not cause specific clinical problems. However, measuring oxygen saturation and erythropoietin levels can be helpful in this regard (7).

When alpha-thalassemia (the absence of one, two, or three genes) is present, the number of α-chain decreases, so in the presence of β-chain hemoglobinopathies, usually due to the higher affinity of the normal β-chain, the HbA level will be higher than the variant hemoglobin. This is not true in the case of β_J_ because due to more negative charge and more tendency to α-chain, the amount of variant hemoglobin will be higher than HbA (7-10). In beta-thalassemia, one of the diagnostic features is the HbA2 elevation. Elevated HbA2 is the body's response to α-chain excess versus β-chain. Globin δ, which is a subunit of HbA2, has a higher positive charge, which slowly forms dimers with α-chain. However, in beta-thalassemia minor, when there is a decrease in synthesis (β^+^) or no synthesis (β^0^), these δ-chains combine with excess alpha and increase HbA2, which is one of the diagnostic indicators of beta-thalassemia minor (9, 10). In case 2 presented earlier, HbA2 increased (4.9%). Because of the patient's thalassemic indices and the absence of HbA in the electrophoresis bands, the absence of β-chain (β^0^) and partnership with β_J_ would be suspected.

Although it is not possible to prove this without molecular tests, the important point in these cases is premarital tests and accurate determination of β genotype (8,10,11). For example, if case 2 has a β_J_/β^0^ genotype and marries a woman with a β/β^0^ or β/β^+^ genotype (beta-thalassemia minor), there is a 25% chance that their child will be born with thalassemia major with possible β^0^/β^0^ or β^+^/β^0^ genotypes. Or if he marries a woman with a sickle cell trait (HbAS or β/β_S_ genotype), there is a 25% chance that the child will suffer from Sickle cell disease with the β_S_/β^0^ genotype, which is very severe and life-threatening anemia. Therefore, it is recommended that the physicians and genetic counselors pay special attention to this possibility and only because β_J_ is not associated with a specific clinical sign, they do not refrain from following and determining the genotype to prevent the birth of a thalassemia major one with pain and the imposition of heavy medical and mental cost. Also, it could be useful to determine Hb J prevalence.
